# A cross-sectional analysis of how young adults perceive tobacco brands: implications for FCTC signatories

**DOI:** 10.1186/1471-2458-12-796

**Published:** 2012-09-17

**Authors:** Philip Gendall, Janet Hoek, Richard Edwards, Judith McCool

**Affiliations:** 1 University of Otago, P O Box 56, Dunedin, New Zealand; 2 University of Auckland, Private Bag, 92019, Auckland, New Zealand

**Keywords:** FCTC, Tobacco branding, Plain packaging, Health policy

## Abstract

**Background:**

The Framework Convention on Tobacco Control calls for the elimination of tobacco advertising, promotion and sponsorship. To test whether tobacco packaging functions as advertising by communicating attractive and distinctive brand attributes, we explored how young adult smokers and non-smokers interpreted familiar and unfamiliar tobacco brands.

**Methods:**

We conducted an on-line survey of 1035 young adult smokers and non-smokers aged 18–30. Participants evaluated eight tobacco brands using ten attributes based on brand personality scales. We used factor analysis and ANOVA to examine patterns in brand-attribute associations.

**Results:**

Young adults distinguished between brands on the basis of their packaging alone, associated each brand with specific attributes, and were equally able to interpret familiar and unfamiliar brands. Contrary to our expectations, non-smokers made more favourable brand-attribute associations than smokers, but both groups described *Basic,* a near generic brand, as ‘plain’ or ‘budget’. There were no significant gender or ethnicity differences.

**Conclusions:**

Tobacco packaging uses logos, colours and imagery to create desirable connotations that promote and reinforce smoking. By functioning in the same way as advertising, on-pack branding breaches Article 13 of the FCTC and refutes tobacco companies’ claims that pack livery serves only as an indentifying device that simplifies smokers’ decision-making. Given this evidence, signatories should see plain packaging policies as a priority consistent with their FCTC obligations to eliminate all tobacco advertising and promotion.

## Background

Internationally, smoking remains the largest cause of preventable death; furthermore, because smoking prevalence is disproportionately high among indigenous peoples and lower socio-economic groups, it contributes to profound health and social inequalities. In the United States, a recent review reported that smoking prevalence varied from 35% among American Indians and Alaskan Natives to 15% among Asian Americans and Pacific Island citizens [1]. Similar discrepancies are evident in Australia, where smoking prevalence among indigenous Aboriginal and Torres Strait Island people is 51% compared to less than 20% among non-Aboriginal people, [2] and in New Zealand, where smoking prevalence among Māori is 44% overall (and over 50% among some groups) compared to 18% among non-Māori [3].

The WHO Framework Convention on Tobacco Control (FCTC), an international treaty with signatories from 168 countries, sets out a plan to reduce smoking prevalence and, in doing so, important health inequalities [4]. Among other measures, signatories to the FCTC have agreed to ban tobacco advertising, promotion and sponsorship, and introduce pictorial warning labels (PWLs) on tobacco packaging. In line with their commitment to Articles 11 and 13, many countries require the removal of tobacco retail displays and the introduction of new PWLs that cover a greater proportion of the pack surface.

The tobacco industry has opposed these measures on the grounds their effectiveness is unproven. More recently, they have fought against plain packaging, which they claim would violate existing trade agreements and misappropriate intellectual property. Industry members or interest groups have lobbied governments, and, in some cases, sued to protect their interests. For example, tobacco companies have secured an injunction delaying the introduction of PWLs in the United States on the grounds these go beyond merely warning smokers, [5] while Philip Morris is suing the Australian government to prevent the introduction of plain packaging, arguing this measure breaches a bi-lateral trade agreement [6].

Internationally, plain packaging proposals have refocused attention on packaging and its role as a communication medium. In line with their FCTC responsibilities, many countries have restricted tobacco marketing and begun considering plain packaging. However, while several studies have documented how plain packaging would decrease smoking’s appeal [7-9], evidence of whether packaging functions as advertising could clarify countries’ FCTC obligations. We address this question by drawing on brand attribute and symbolic consumption theory to explore how young adult smokers and non-smokers interpret tobacco packages.

### Brand attributes and symbolic consumption

Marketers rely on branding to associate aspirations, attributes and values with functional products and services. The resulting relationships mean consumers buy branded products as much for their symbolic value as for their utility [10-12]. Repeated pairing of branded products with positive contexts, colors and symbols creates favorable and brand-specific connotations, to the point where a brand alone eventually evokes those associations and the benefits assumed to follow [13]. The associations and images physical brand insignia connote have become critical points of differentiation for tobacco products, which rely heavily on emotional and symbolic attributes to attract new users [14].

Consumers use physical brand attributes to construct imagery that they draw on and personalize; ultimately, brands help consumers to co-create an identity they project to others [15]. Known as symbolic consumption, this process involves consumers forming relationships with brands, which they use to structure and create meaning in their lives [16]. As a result, tobacco manufacturers sell status, social acceptance, glamour and adventure, rather than simply a device to deliver nicotine [14,17,18]. Internal tobacco industry documents reflect a deep understanding of symbolic consumption and reveal meticulous research into pack designs, brand insignia, and the images consumers create using these [14,16,19-21]. Furthermore, they highlight the importance the tobacco industry places on young adult smokers and brands that appeal to this group’s uncertainties and aspirations.

Young people’s use of brand imagery to shape their public personae first stimulated interest in plain packaging nearly two decades ago when Canadian and New Zealand researchers independently examined how young people perceived plain packaging [22,23]. Researchers reported that young people had consistently more negative impressions of plain packs relative to branded cigarette packs [24,25]. More specifically, respondents regarded plain packages as old fashioned and boring, and thought fewer people would smoke if cigarettes were sold in plain packages. The researchers concluded that reducing on-pack brand insignia would diminish the physical and social attractiveness of tobacco products, promote cessation among some smokers, and reduce initiation among those experimenting with tobacco.

Recent experimental studies have also found that smokers strongly prefer branded packs to plain packs, and concluded that plain packs would be more likely to stimulate cessation-related behaviors [7-9]. In addition, plain packaging reduces ambiguity created by pack colors, which tobacco companies have used to suggest some variants pose fewer risks to smokers than others [26-28].

Despite evidence that plain packaging would reduce smoking’s appeal, few researchers have examined whether tobacco packaging functions as advertising by communicating positive brand attributes. Earlier qualitative studies exploring young people’s responses to plain packaging were undertaken when most jurisdictions still permitted tobacco advertising in mass media, and prior to the introduction of pictorial warnings. Although experimental studies show participants’ perceptions of smoking decline as they are exposed to packs with fewer brand elements, these findings do not establish that packaging functions as advertising. Studies using branding theory to explore whether and how young adults interpret on-pack imagery could address this evidence gap and strengthen calls for plain packaging.

Evidence that young adults’ perceptions of cigarette brands vary suggests packaging functions as an advertising medium and could strengthen calls for plain packaging measures. By contrast, if young adults see tobacco packages as largely indistinguishable, we could not conclude that packs function as an advertising medium. Given tobacco industry documents revealing companies’ careful research into brand imagery and its effects, we hypothesized that:

H_1_: Young adults will associate different attributes with different tobacco brands and differentiate between these solely on the basis of their packaging.

As users of a product category have greater knowledge of the brands within that category, we also hypothesized that:

H_2a_: Young adult smokers will have a higher level of brand attribute associations, and more positive associations, with familiar brands than will young adult non-smokers.

H_2b_: Young adult smokers will have a higher level of brand attribute association, and more positive associations, with unfamiliar brands than will young adult non-smokers.

Finally, if plain packaging reduces tobacco brands’ ability to function as an advertising medium, we hypothesized that:

H_3_: Young adult smokers and non-smokers will not associate positive attributes with a largely generic brand.

## Methods

The New Zealand Health Research Council (HRC) funded the study, which underwent a full ethics review and approval process (known as Category A approval) at the University of Otago (approval number 09/ 165) Formal consultation was also undertaken with the Ngai Tahu Consultative Committee.

The study involved a survey of 1035 New Zealanders aged between 18 and 30 from an online consumer panel provided by SmileCity. SmileCity is one of the largest market research panels in New Zealand, with an active panel of 223,527 as at May 2011. The survey was conducted between 16 September and 5 October 2011 and quotas were applied to ensure adequate representation by gender (408 males vs 627 females), ethnicity (Māori and Pacific Island 594 vs non-Māori/other 441) and smoking status (smoker 485 vs non-smoker 550).

Respondents were presented with images of seven cigarette brands: *Holiday, Basic, Camel No. 9, Merit, Port Royal, Kool* and *Longbeach.* Three of these brands – *Holiday* (mid-price tailor made cigarettes), *Longbeach* (lower price tailor made cigarettes) and *Port Royal* (low price loose tobacco) – are available in New Zealand and represent brands with high (*Holiday*) and low (*Longbeach*) penetration among young adult smokers [29]. Apart from *Camel No. 9*, which some respondents may have heard of as the parent brand *Camel* (which is available in New Zealand), respondents were unlikely to have encountered the remaining four brands, which were US tobacco brands with varied penetration levels in markets outside New Zealand. The *Basic* brand, as its name suggests, is largely generic and has few brand elements; it functioned as a control relative to the other clearly branded packs.

**Figure 1:**
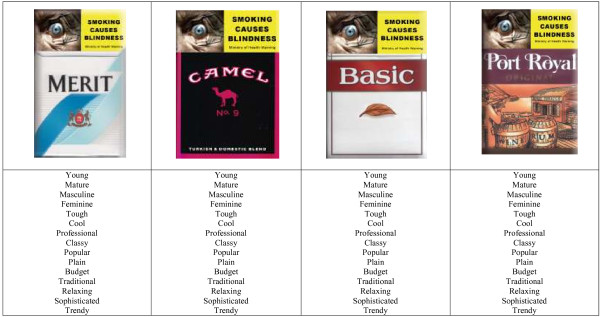
Examples of Test Stimuli and Attributes.

Respondents used 15 adjectives selected to correspond to various brand personality dimensions [10] and that we had pre-tested in earlier studies assessing tobacco product positioning [9,30]. These included: young, mature, masculine, feminine, tough, cool, professional, classy, popular, plain, budget, traditional, relaxing, sophisticated, and trendy. Respondents were asked to associate as many or as few of these 15 attributes with each of the seven brands, depending on their perception of the brand concerned. The order of presentation of both the brands and the attributes was randomised to avoid question-order and item-order effects. Figure 1 outlines the question used and contains examples of the brand stimuli and attributes.

### Analysis

All analyses were undertaken using PASW(18). We initially used Principal Components Analysis to examine each brand’s underlying attributes and then ANOVA to test differences between gender, ethnicity and smoking status. Although we detected some differences for gender and ethnicity, these were not systematic. The only consistent differences occurred between non-smokers’ and smokers’ responses; the results section thus focuses on these groups, while noting differences by other variables where relevant.

## Results

Most individual respondents selected between one and four attributes per brand; the mean number ranged from 1.7 to 2.5 and the median for all seven brands was two attributes. On average, each of the 15 attributes was associated with a particular brand 144 times, with a range between 0 and 705 associations.

The first hypothesis posited that each tobacco brand would communicate different attributes to young adults. To test this hypothesis, we first examined the *Basic* brand, which we used as a control. Given its generic appearance and name, it is not surprising that virtually the only attributes associated with *Basic* were ‘plain’ and ‘budget’. For this reason, we excluded *Basic* from further analysis and discuss its evaluation separately. We then factor-analysed the brand descriptors for the remaining six brands. These analyses produced between three and five significant factors (Eigen values greater than 1.00) for each brand. Table 1 contains an example of the factor analysis results for a familiar brand (*Port Royal)*.

**Table 1:** Rotated Component Matrix: Port Royal

	**Component**				
	**1**	**2**	**3**	**4**	**5**
Popular	.736				
Cool	.627				
Relaxing	.566				
Trendy	.525				
Sophisticated		.731			
Classy		.661			
Feminine					
Tough			.796		
Masculine			.725		
Budget				.790	
Plain				.746	
Traditional					.808
Mature					.609

Extraction Method: Principal Component Analysis.

Rotation Method: Varimax with Kaiser Normalization.

**Figure 2:**
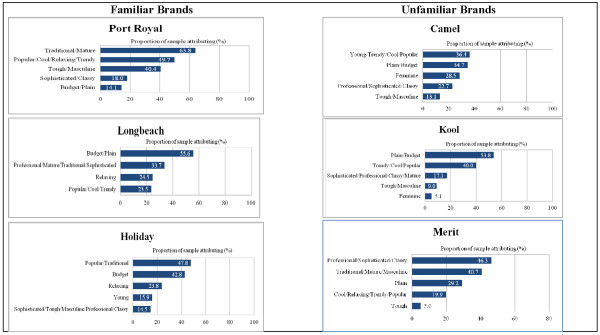
Perceived Attributes of Familiar and Unfamiliar Brands.

After separating ‘plain’ and ‘budget’ from the positive attributes of the factors on which they loaded for other brands and treating these independently (to enable comparisons with *Basic*), five underlying constructs emerged for each brand, except for *Longbeach*, which had four. As Figure 2 illustrates, the particular constructs on which individual attributes loaded, as well as the relative importance of each construct, varied considerably between brands.

Among familiar brands, participants saw *Port Royal* as primarily traditional and mature, popular and relaxing, and masculine; very few regarded it as a sophisticated brand, though neither did they consider it budget or plain. Analyses by ethnicity showed that male Māori and Pacific respondents saw the brand as less traditional/mature than did other groups, particularly other male smokers, and female Māori and Pacific smokers. Most participants regarded *Longbeach* as a primarily plain and budget brand, an association that reflects its lower price point. However, more than a third also saw it as professional and mature, and a quarter considered it relaxing, and popular and trendy. Māori and Pacific were more likely to make these latter associations than non-Māori and Pacific. Participants also saw *Holiday* as popular and traditional (particularly by women), and budget (again, a reflection of the brand’s lower price position).

Participants associated fewer attributes with *Camel No.* 9, a brand variant not sold in New Zealand. However, many regarded it as a younger brand, likely to be sold at a lower price point, and more likely to be targeted at women than men. Respondents saw *Kool* as a primarily plain or budget brand, but also associated it with trendy and cool attributes, and saw it as lacking a specific gender appeal. Of the three unfamiliar brands tested, *Merit* had strong associations with professionalism and maturity, and was seen as a traditional, older brand.

Overall, these findings support our first hypothesis: respondents associated different attributes with the test brands, which each had a distinctive profile. Although the brands had similar underlying constructs, the proportion of participants associating these with each brand varied and suggested considerable diversity in their overall perceptions. Aside from *Camel No.* 9, which had slightly fewer attribute associations than other brands, participants did not associate more attributes with familiar than unfamiliar brands.

**Figure 3:**
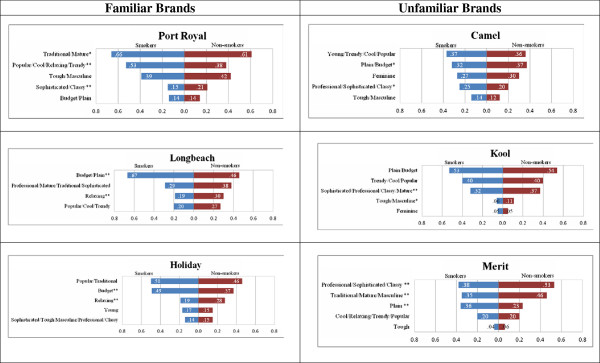
Brand Attribute Associations by Smoking Status.

To test the second hypothesis, we conducted analysis of variance on the proportion of the sample that attributed a construct to the brand concerned; these analyses compared the associations made by smokers and non-smokers. Figure 3 contains these findings.

Overall, non-smokers were often significantly more likely to associate positive attributes, such as ‘sophistication’ with familiar brands than were smokers, who often saw the brands as ‘budget’. For example, more smokers associated the words ‘budget’ or ‘plain’ with *Longbeach* and *Holiday,* while more non-smokers saw these brands as ‘sophisticated’, ‘trendy’, ‘relaxing’ or ‘professional’. However, smokers were significantly more likely than non-smokers to see *Port Royal* as popular and cool, though slightly less likely to consider it sophisticated.

These findings illustrate how packaging alone communicates positive associations, even to people with no current experience of the product category. They do not support H2a, which adopted the tobacco industry’s argument and posited that smokers, familiar with the product category, would make more brand associations and associate more positive attributes with brands.

We expected smokers and non-smokers to have similar levels of attribute association with less familiar brands. However, non-smokers were generally more likely to make positive associations than smokers. For example, they were significantly more likely to link *Merit* with the professional/sophisticated/classy construct, and less likely to regard it as plain. Non-smokers were also more likely to associate these attributes with *Kool*. Only *Camel No. 9* reflected the hypothesized pattern (similar attribute association by smokers and non-smokers) and, as noted earlier, it had a lower overall level of attribute association than other brands.

These findings do not support hypothesis 2a or 2b. Whereas we expected smokers to make more positive attribute associations with familiar brands, they were actually more likely to see these brands as budget and less likely to regard them as relaxing than non-smokers. Although we expected smokers and non-smokers to respond in similar ways to unfamiliar brands, in fact non-smokers associated more positive attributes with these than did smokers. The results illustrate how tobacco packaging communicates positive attributes to non-smokers and question industry claims that on-pack branding serves only as an indentifying device that simplifies smokers’ decision-making. Non-smokers’ perceptions of the unfamiliar brands suggest on-pack imagery creates attractive ‘personalities’ that appeal to different potential consumer segments, and that may encourage and stimulate smoking experimentation.

**Figure 4:**
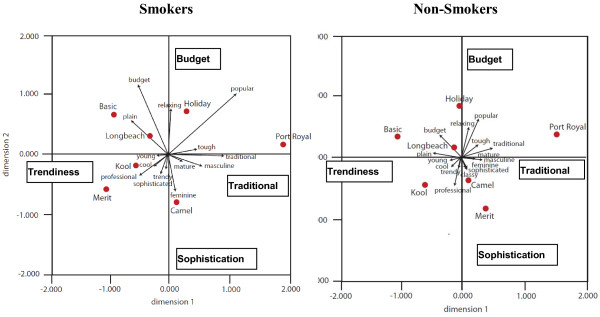
Smokers’ and Non-smokers’ Brand Perceptions.

As both Figures 1 and 2 illustrate, the attributes often loaded on different dimensions for each brand. Thus, while ‘traditional’ and ‘mature’ loaded on the primary factor for Port Royal, ‘popular’ and ‘traditional’ loaded on the main factor for Holiday. Like individuals, brands’ personalities sometimes appeared potentially contradictory and participants saw brands as possessing ostensibly inconsistent attributes. Figure 4 illustrates the overall brand maps for both smokers and non-smokers and reinforces how both groups associated the brands with distinct sets of attributes.

The perceptual maps in Figure 3 illustrate the test brands’ positions in the relevant product spaces. Dimension 1 is anchored by tradition and trendiness while Dimension 2 reflects the budget attribute and sophistication. Participants saw brands located in the top left quadrant as plain and budget, while those in the top right quadrant were more traditional and popular. Brands in the lower left quadrant appeared more youthful for non-smokers (though smokers placed *Merit* in this space) while those in the lower right were generally seen as more sophisticated.

**Figure 5:**
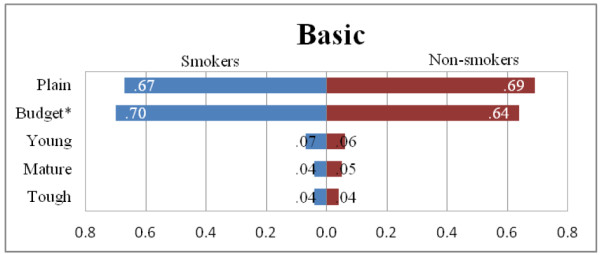
**Brand Attribute Associations for *Basic*.**

Our final hypothesis examined attribute association with a largely generic brand: *Basic*. Participants associated only the ‘plain’ and ‘budget’ attributes with *Basic*; smokers and non-smokers were equally likely to describe it as plain and smokers were slightly more likely to regard it as budget (see Figure 5).

These results demonstrate how participants see a brand featuring virtually no imagery as lacking any redeeming connotations, a finding that suggests truly dissuasive packaging may create negative connotations about smoking.

## Discussion and conclusions

Brand association and symbolic consumption theory highlight the importance of logos, colours and imagery, which combine to create aspirational values and desirable connotations that consumers access when they consume and display a brand [15,31]. Because branding promotes products’ symbolic values, it has particular appeal to the tobacco industry, which can no longer access many traditional marketing media. Thus, tobacco companies have invested heavily in package design; their brands have high strategic value because they enable continued promotion of specific brand attributes [14,18].

However, the FCTC requires signatories to eliminate tobacco advertising and marketing. Regulation of tobacco packaging’s pivotal marketing role, particularly in ‘dark’ markets that restrict other marketing media, has led policy makers to propose (or consider proposing) plain packaging [32]. This measure is opposed as strongly by tobacco companies as it is supported by public health researchers.

This study contributes to the debate over plain packaging by examining what, if any, attributes familiar and unfamiliar tobacco brands communicate to young adults, and how smokers and non-smokers interpret tobacco brands. Young adults distinguished between brands on the basis of their packaging alone, recognised a clear ‘personality’ for each brand, and were equally able to interpret familiar and unfamiliar brands.

If, as the tobacco industry has argued, branding serves only to promote brand-switching among existing users, smokers should have associated more attributes (and more favourable attributes) with familiar brands. However, non-smokers often made more favourable brand-attribute associations than did smokers. While the data do not enable further probing of this intriguing finding, other studies have documented high levels of regret among smokers, [33,34] which may have translated into less positive evaluations of tobacco brands. Future research could explore this ambivalence and the factors responsible for it.

Smokers and non-smokers also differed in their perceptions and appraisals of unfamiliar brands, which non-smokers often associated with more positive attributes, and were less likely to see as ‘plain’. These findings challenge the industry’s stance and suggest on-pack branding may communicate even more effectively to non-smokers than smokers, thereby increasing non-smokers’ susceptibility to smoking and facilitating their experimentation.

Evidence that neither smokers nor non-smokers saw the *Basic* brand as anything other than ‘plain’ or ‘budget’ also indicates how largely generic packaging can remove the positive associations created by branding. This finding supports conclusions from experimental studies documenting the decreased attractiveness of plain packaging [7-9], and suggests this measure will elicit strong negative attribute associations that will reduce the cachet of smoking and may deter experimentation.

We did not test whether plain packaging will reduce smoking prevalence, but our findings illustrate how packaging communicates brand attributes, which earlier studies and industry documents show appeal strongly to young people [19-21,25]. Non-smokers’ ability to access, interpret, and understand tobacco brand attributes with apparent ease suggests on pack-branding functions as advertising and implies that implementation of FCTC Article 13 requires signatories to implement plain packaging.

Our study builds on exploratory work and uses a large and diverse sample to examine brand-attribute associations; nevertheless, it has some limitations. Of these, the most important is that brand-attribute associations alone do not indicate increased risk propensity; nor, as noted above, do they demonstrate that plain packaging would reduce smoking prevalence. Further experimental work carefully testing the appeal and likely response to packs with varying levels of branding will be required to address this limitation.

Notwithstanding this caveat, the findings extend our understanding of how young adults understand tobacco branding. Bearing in mind that participants reviewed the brands quickly and were unlikely to have engaged in detailed or systematic processing, their widely varying brand-attribute associations highlight the evocative nature of tobacco branding. We found few differences by ethnicity and gender, and no systematic patterns within the data for these characteristics. Only smokers and non-smokers varied in their brand attributions, and then not in the direction that would support the tobacco industry’s claims. This result suggests plain packaging would be widely seen as ‘budget’ and ‘plain’ by young adults, the group at greater risk of initiation, and subsequent addiction and harm.

Further evidence that tobacco packaging functions as advertising challenges FCTC signatories, who have pledged to remove tobacco advertising and promotion, to move rapidly towards mandating plain packaging. Given the growing evidence of packaging’s crucial role as a marketing medium, policy makers have few reasons to delay the introduction of plain packaging.

## Competing interests

We have no financial or non-financial competing interests. However, for the sake of full disclosure we note that this study was funded by the Health Research Council of New Zealand (grant 09/195R). JH and RE have received funding for tobacco control research from the Royal Society of New Zealand (Marsden Fund) and the Cancer Society of New Zealand. PG, JH and RE have received funding for tobacco control research from the New Zealand Ministry of Health. All authors have received funding for tobacco control research from the Heart Foundation of New Zealand.

## Authors’ contributions

JH conceived of the study and with PG designed and pre-tested the survey; PG developed the survey website and analysed the data. JH and PG led the MS development. RE and JM provided feedback on the instrument and data interpretation, and contributed to the final MS. All authors have read and approved the final MS.

## Pre-publication history

The pre-publication history for this paper can be accessed here:

http://www.biomedcentral.com/1471-2458/12/796/prepub
